# Functions of the Proteasome on Chromatin

**DOI:** 10.3390/biom4041026

**Published:** 2014-11-21

**Authors:** Tyler S. McCann, William P. Tansey

**Affiliations:** Department of Cell and Developmental Biology, Vanderbilt University School of Medicine, 465 21st Avenue South, Nashville, TN 37232, USA; E-Mail: tylersmccann@gmail.com

**Keywords:** chromatin, transcription, gene regulation, proteasome, proteolysis, chaperone

## Abstract

The proteasome is a large self-compartmentalized protease complex that recognizes, unfolds, and destroys ubiquitylated substrates. Proteasome activities are required for a host of cellular functions, and it has become clear in recent years that one set of critical actions of the proteasome occur on chromatin. In this review, we discuss some of the ways in which proteasomes directly regulate the structure and function of chromatin and chromatin regulatory proteins, and how this influences gene transcription. We discuss lingering controversies in the field, the relative importance of proteolytic *versus* non-proteolytic proteasome activities in this process, and highlight areas that require further investigation. Our intention is to show that proteasomes are involved in major steps controlling the expression of the genetic information, that proteasomes use both proteolytic mechanisms and ATP-dependent protein remodeling to accomplish this task, and that much is yet to be learned about the full spectrum of ways that proteasomes influence the genome.

## 1. Introduction

One of the most important tasks of any normal cell is to maintain the proper integrity and expression of its genetic information. Alterations to the genome or the transcriptome can lead to cell death or cancer, and cells possess a battery of processes to ensure that DNA is appropriately packaged, expressed, repaired, and duplicated. In recent years, it has become clear that one of the processes that cells frequently use to control and drive chromatin-dependent events is the ubiquitin (Ub)-proteasome system (UPS). Ubiquitin and the proteasome have been implicated in processes as diverse as the control of transcription, the response to DNA damage, the regulation of chromatin structure and function, and export of RNAs from the nucleus, and a number of excellent reviews have covered the involvement of Ub-dependent events in these processes (e.g., [1,2,3,4,5]). What we would like to do here, however, is focus specifically on the functions of the proteasome in the context of chromatin. Although it is difficult to divorce proteasomes from ubiquitin, chromatin and transcription is a venue in which non-canonical functions of the proteasome have been proposed and documented, and in which considerable controversy has brewed regarding how proteasomes interact with chromatin and which functions of the proteasome are important for controlling events such as transcription. Recent work in this area has shed light on proteasome action in this context, and it is now possible—with the benefit of hindsight—to reflect on observations that have been made in this area over the last 20 years and distill some general themes in how proteasomes act on chromatin. The purpose of this review, therefore, is to discuss some of the more prominent ways that proteasomes control transcription and chromatin, offer some insight that rationalizes seemingly disparate observations, and highlight areas that are ripe for exploration in the future.

## 2. A Primer on Chromatin

The typical mammalian cell carries roughly two meters of DNA—billions of base-pairs—that needs to be neatly packaged within a nucleus less than 20 micrometers in diameter [6]. This ultimate storage solution is achieved by the hierarchical compaction of DNA with histones; first with two copies each of the four core histones (H2A, H2B, H3, and H4) to form the nucleosome, then between nucleosomes and histone H1 to form the 30 nm fiber, and then between 30 nm chromatin fibers in successive iterations to form the chromosome. Packaging of DNA into chromatin not only allows the genetic information to squeeze into the tight nuclear space, but is essential for the safe passage of replicated DNA to daughter cells, for coordinating critical events in genome maintenance and repair, and for proper control of gene expression [7].

The regulatory impact of chromatin has come into sharp focus over the last twenty years, hand-in-hand with a deeper understanding of the epigenetic mechanisms that preside over gene expression [8]. Although the ways in which chromatin controls DNA-centric events are many, the founding principle connecting all of these processes is the notion that tight packaging of DNA with histones restricts the availability of DNA, requiring that chromatin be decondensed and nucleosomes dismantled or reorganized to replicate, repair, or transcribe a particular segment of the genome. Additionally, because proteins are inherently more complex than nucleic acids, incorporation of DNA into an ordered mass with proteins allows chromatin to integrate a myriad of signaling processes to control access to the DNA, or to signal to the cell that a specific piece of DNA is damaged, recently transcribed, or available for transcription in the future.

A full discussion of the influence of chromatin on DNA-dependent processes is beyond the scope of this review, but a few salient points are worth making here. First, the events of chromatin assembly and disassembly occur at multiple levels of nucleosome organization, and are active, enzyme-mediated, processes. A suite of remodeling enzymes and histone chaperones [9] exist that drive transitions in chromatin structure, moving nucleosomes to expose or conceal regulatory DNA elements, evicting nucleosomes ahead of RNA polymerases and reassembling them in their wake, or exposing damaged DNA segments for refurbishment. The involvement of enzymes in these events confers tremendous regulatory potential to DNA-dependent transactions and permits the proper sequencing of multistep biological reactions needed to express or repair the genetic material.

Second, histones are subject to a huge assortment of post-translational modifications—including phosphorylation, acetylation, methylation, ubiquitylation, and SUMOylation [10]—that profoundly influence genome behavior. These modifications can alter the physical properties of chromatin to enhance or suppress transcription [11], recruit chromatin modifiers and other regulatory proteins to specific sites in the genome [12], signal the presence of DNA damage [13], and establish chromatin domains as active (euchromatin) or silent (heterochromatin), among other things. The rich regulatory repertoire of histones is further enhanced by the fact that these modifications can influence each other and work combinatorially to modulate chromatin function [8], and by the existence of variant histone proteins [14] that are incorporated into chromatin at specific times and places to alter its biological properties. Histone modifications and variants are a major source of epigenetic phenomena, and profoundly shape transcriptional patterns and responses in eukaryotic cells.

Finally, and implicit in the above discussion, chromatin is a highly dynamic entity. It is constantly being built, deconstructed, and rearranged in response to transcription, DNA-repair, and during the events of cell duplication and division. Additionally, post-translational modifications on the histones are in a constant state of flux, and just about every enzyme that “writes” these modifications is countered by another that will reverse, or “erase” the process. Cells employ a tremendous amount of resources to dynamically warehouse their DNA, but the net effect of this dynamism—together with the inherent diversity of histone remodelers, variants, and modifications—is to create a balance between maintaining essential genomic functions required for cell identity and viability, while at the same time creating continuous opportunities for cells to modulate their genomic functions as the need arises.

## 3. The Proteasome and Chromatin: A Caveat

As the proteolytic end-point in Ub-mediated proteolysis, the proteasome has the potential to directly or indirectly control the steady-state level of just about every cellular protein. Any discussion of its actions in a particular process, therefore, has to distinguish between direct involvement of the proteasome in regulating key biochemical steps in that process and indirect effects where the proteasome is simply determining whether proteins generally accumulate in the cell and at what level. In the absence of biochemical reconstitution of particular processes, it is difficult to rigorously discriminate between these possibilities, and this is a general limitation in our understanding of how the proteasome regulates genomic events, where defined reconstitution experiments are few, and where many of the genetic and biochemical tools used to interrogate proteasome function in this context have broad effects on a host of cellular activities [1].

With that caveat in mind, however, there is good reason to believe that proteasomes are involved in proteolytic and non-proteolytic events that occur directly on, or in the immediate vicinity of, chromatin and impact genomic events in a direct and mechanistic way. Historically, it is worth remembering that the first ubiquitylated protein to be discovered was histone H2A [15], that histone H2A ubiquitylation was shown as early as 1982 to be enriched on active chromatin [16], and that proteasome particles were observed in mammalian nuclear extracts at around the same time [17]. It is also worth noting that some of the first compelling genetic data tying proteasome function to gene expression predates biochemical characterization of the proteasome [18], meaning that links between the proteasome and chromatin-dependent processes have been recognized for almost as long as contemporary understanding of the UPS itself. It is now clear that proteasome components are present in the nuclei of actively-dividing eukaryotic cells [19], associated with chromatin [20], and enriched at specific sites in the genome [21,22,23,24,25] and in response to specific molecular events such as transcription [21] or DNA damage [26]. Moreover, inhibition of proteasome function results in profound changes in the distribution of ubiquitylated proteins on chromatin [27], implying that Ub-mediated proteolysis most likely occurs within the immediate confines of the chromatin environment in which these proteins act. Thus, although it is not always possible to directly and unambiguously tie proteasomes to the biochemical operations of chromatin, strong circumstantial evidence places proteasome subunits and their activities at “the scene of the crime”. Unless otherwise noted, our review focuses on examples where physical or functional evidence implies a direct role for chromatin-associated proteasomes in genomic processes, or where the most likely interpretation of studies is that proteasomes are acting locally to specifically control chromatin-based phenomena.

## 4. The Form of the Proteasome That Associates with Chromatin

The term “proteasome” conjures an image of the archetypal 26S complex, with a barrel-like 20S core capped at one or both ends by a 19S regulatory particle [28]. Although this view is oversimplified, and alternative proteasomes do exist (e.g., [29,30,31]), in most biological settings it is generally assumed that canonical 26S proteasomes, or slight variants thereof, are at work. Not so when it comes to transcription, chromatin, and the proteasome. Indeed, the basic issue of the form of the proteasome that is recruited to chromatin is steeped in controversy fueled by seemingly contradictory reports on which subunits of the proteasome can associate with chromatin and whether proteolytic or non-proteolytic proteasome activities are required for transcriptional processes.

Tracking how proteasome components physically interact with specific segments of chromatin is most commonly done via chromatin immunoprecipitation or ChIP. In this technique, cells are treated with crosslinking reagents to “freeze” macromolecular interactions, chromatin is isolated, and proteins of interest recovered by immunoprecipitation—along with the DNA with which they were associated in the cell. After crosslinks are reversed, the DNA can be detected by a number of methods such as PCR, for looking at specific sites, or microarrays/next-generation sequencing for gathering an entire genome’s worth of information (e.g., ChIP-seq [32]).

ChIP experiments exploring the interaction of proteasome subunits with chromatin have produced contradictory results in terms of how 19S *versus* 20S subunits behave. Some studies have reported identical or overlapping patterns of binding for 19S and 20S proteins [21,23,26,33,34,35], others have focused specifically on 19S components [25,36,37,38,39,40,41], and others still have reported that 19S and 20S proteasome subunits behave differently in terms of chromatin association patterns, with significant disparity in ChIP signals of 19S *versus* 20S proteins [24,42]. At the activated *GAL1* gene, for example, Gillette *et al.* [42] reported that 19S proteins associate with the *GAL1* promoter (5') soon after induction, absent of any 20S proteins, whereas 20S proteins associate later in the induction process and accumulate at the 3' end of the gene. The marked disparity in these findings makes it impossible to know whether it is the proteasome *per se* that is broadly recruited to chromatin or whether distinct proteasome subassemblies—or entirely new complexes containing select proteasome parts—are recruited for non-proteolytic actions on the chromatin template.

What is the cause of this confusion? One possibility is that proteasome subunits are heterogeneous in how they associate with chromatin, and that proteasome subassemblies are separated or remodeled specifically for their involvement in genome maintenance activities [43]. A simpler explanation, however, is that limitations of the ChIP technique lie at the heart of these discrepancies. Epitope-tagging of proteasome subunits is widely used in yeast to monitor chromatin interactions by ChIP, but such tagging events are not always neutral, and can disrupt the kinetics of *GAL* gene activation in yeast [21]. Moreover, issues of antibody properties and epitope accessibility make it impossible to rigorously interpret negative results, and to conclude, for example, that 20S proteins are *not* present at a particular site on the genome. Supporting the notion that the ChIP technique is the source of this confusion, analysis of a panel of ChIP-validated antibodies against untagged subunits from the 19S lid, 19S base, and 20S core revealed identical temporal and spatial distribution at all sites examined [21], including at *GAL* genes where profound differences were reported earlier [42].

Given concerns with ChIP, and the absence of stringent biochemical characterization of chromatin-dedicated proteasome subassemblies, we suggest that the form of the proteasome that associates with chromatin is the canonical 26S complex. Rigorous support for this notion will require more extensive analysis of additional subunits, genome-wide approaches using antibodies against native subunits, and application of different techniques (e.g., [44]) to monitor proteasome-chromatin interactions in living cells. But in the interim, viewing 26S proteasomes as the genome-relevant species reconciles much of the available information, and allows new predictions to be made in terms of how proteasomes are recruited to chromatin and which biochemical functions are at work.

## 5. Proteasome Location and Recruitment

If we assume that positive localization of one or more proteasome subunits on chromatin reflects the presence of the entire 26S complex, where, when, and how are proteasomes recruited to the genome?

**Figure 1:**
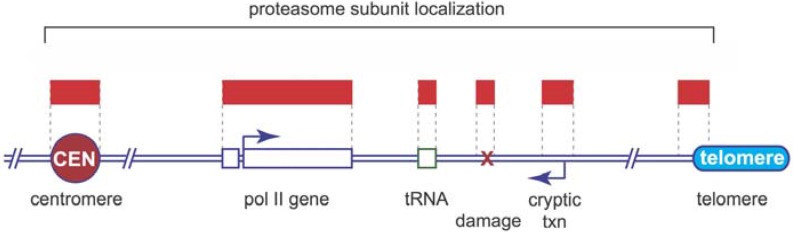
Localization of proteasomes on chromosomes. The cartoon shows an idealized eukaryotic chromosome, depicting the centromere (“CEN”) a pol II-transcribed gene, a tRNA gene, a site of DNA damage, a site of cryptic transcription (“cryptic txn”) and a telomere. Above the cartoon, symbolized with red bars, are regions shown to be bound by one or more proteasome subunits.

Overall, proteasomes appear to be widely associated with chromatin, and in a manner that reflects activity occurring at specific sites in the genome (Figure 1). On an individual gene level, proteasomes are rapidly recruited to the RNA-polymerase II (pol II)-transcribed *GAL10* gene after its induction, with kinetics mirroring that of pol II itself [21]. Once induced, proteasomes associate with the entire transcribed portion of *GAL10*, and in a manner that is dependent on ongoing transcription, as proteasome subunits disperse rapidly after transcription is shut down [21,33]. Interestingly, this phenomenon is not restricted to pol II-transcription, as tRNA genes transcribed by RNA polymerase III (pol III) are also proteasome-associated; again in a transcriptionally-dependent fashion [21]. More broadly, genome-wide studies have reported that one or more proteasome subunits are found at the majority of transcriptionally-active loci in yeast [23,33] revealing a comprehensive involvement of the proteasome (in some way) in the majority of transcriptional events that occur in a eukaryotic cell. Taking these observations together, we speculate that proteasomes are recruited to chromatin in response to dynamic changes that occur commensurate with transcription, and that they participate in processes that occur not just at one step in pre-mRNA synthesis, but along the entire gene.

Besides bona fide transcriptional units, proteasome subunits have also been spotted at sites of cryptic (specifically intergenic) transcription in mouse embryonic stem (ES) cells [35], a budding yeast telomere [21], a fission yeast centromere [25], and to a site of induced, double-stranded DNA breaks [26]. The widespread appearance of proteasomes on different functional types of chromatin further suggests that its activities are not limited to regulating one specific protein or process, but rather being commandeered to impact multiple steps in accessing, modifying, or controlling chromatin.

**Figure 2:**
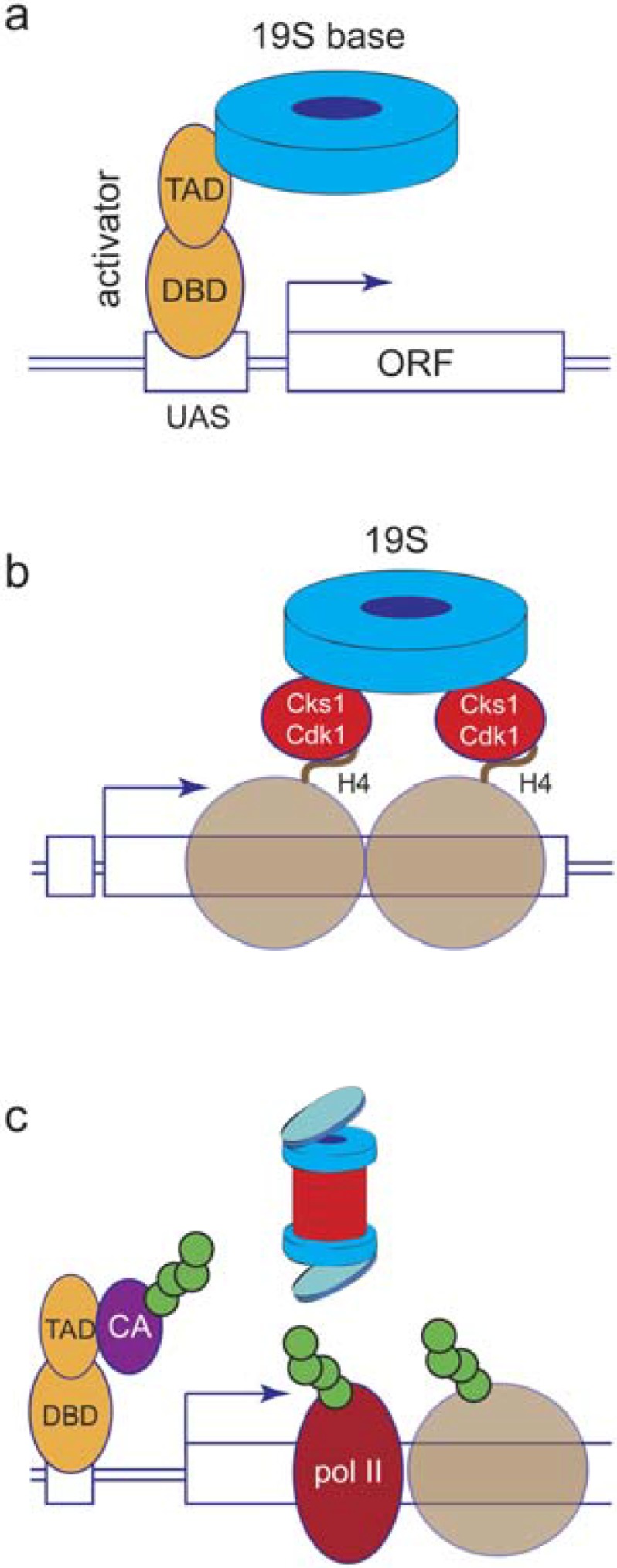
Models for recruitment of proteasome subunits to chromatin. (**a**) Recruitment by direct contact with activators. A model activator, with its separable DNA-binding domain (DBD) and transcriptional activation domain (TAD) is shown bound to an upstream activating sequence (UAS) at a yeast promoter. In this model, direct contact between the TAD of the activator and one or more ATPases in the 19S base complex leads to promoter-selective recruitment of 19S base components to chromatin; (**b**) Recruitment by intermediary proteins. In this model, binding of an adapter complex (consisting of Cks1 and Cdk1) to histone H4 (brown circle) tails leads to recruitment of 19S proteasome components; (**c**) Recruitment by ubiquitylated substrates. In this model, we propose that the presence of one or more ubiquitylated substrates on chromatin recruits proteasomes. “CA” stands for co-activator. Note that this model is consistent with canonical views of how the proteasome is recruited into other biological processes, but the specific ubiquitylated proteins here are arbitrary.

The rather specialized places that proteasome subunits appear on chromatin supports the concept that proteasomes are actively recruited to specific regions of the genome in response to one or more molecular signals (Figure 2). One school of thought posits that proteasomes are recruited via direct interactions with select chromatin-bound proteins, such as the Gal4 activator [24,45] and nucleosomal histones [46,47]. Indeed, there is compelling evidence that proteasomes present in mammalian testes (“spermatoproteasomes”) contain 20S complexes capped by the alternative activator PA200/Blm10, and that PA200/Blm10 carries bromodomain-like regions capable of directly binding to acetylated histone tails [47], permitting *Ub-independent* degradation of core histones during spermatogenesis. Although such processes could certainly recruit proteasomes in specific instances, mechanisms such as these cannot account for the number and diversity of proteasome-association sites in genome.

An alternative model for how the proteasome is recruited to chromatin, and one that reconciles how proteasomes are able to interact with so many different loci, is that proteasomes simply come to chromatin in response to the presence of ubiquitylated proteins at these sites. In this model, the precise ubiquitylated protein(s) could be different at each site (or type of sites), but the ultimate effect is the same—to attract proteasomes to those sites. This notion is aligned with the concept that 26S proteasomes are the chromatin-relevant species, and supported by a recent study from the Scadden laboratory that mapped degradative ubiquitylation sites in human chromatin [27]. Specifically, Scadden and colleagues showed that proteasome inhibition results in the accumulation of chromatin-associated ubiquitin in the 5' regulatory regions of highly active genes, implying that Ub-conjugates normally present at these sites are being degraded by the proteasome. Although there is no guarantee that Ub-conjugates will universally accumulate upon proteasome inhibition, these results strongly support the idea that proteolytically-active proteasomes (*i.e.*, 26S) engage sites of transcription to process ubiquitylated substrates. Importantly, parallel ChIP-seq with the 20S β1 subunit revealed identical patterns of genomic distribution with ubiquitin, including a significant redistribution to sites of ubiquitin accumulation upon proteasome inhibition. It seems very likely, therefore, that much of the association of proteasomes with the genome is driven by active ubiquitylation of chromosome-bound proteins. If correct, this realization makes identification of these—as yet unknown—ubiquitylated proteins key to understanding how proteasomes are mustered to chromatin.

A final issue regarding the prospect that ubiquitylated substrates recruit proteasomes to chromatin is whether proteasomes themselves could access and process target proteins that are engaged with the DNA template. Recent evidence suggests that proteasomes may not be able to accomplish this task alone, but sometimes require a poly-ubiquitin-selective, ATP-dependent, segregase complex known as Cdc48 (p97 in mammalian cells) to extract substrates prior to degradation. Cdc48-dependent extraction of ubiquitylated substrates from chromatin has been shown to occur with the yeast MATα2 repressor [48], pol II after DNA damage [49], and with both natural and synthetic transcriptional regulators [50], suggesting that many, if not all, DNA-bound substrates need to be passed through Cdc48 complexes to reach the proteasome. An intermediary role for Cdc48 in such events does not detract from the importance and immediacy of proteasome function in chromatin-centric events, but it does expose another potential point of regulation in such processes and highlights the need for comparative analyses of proteasome and Cdc48 subunit distribution and involvement in transcription and other biochemical activities that are based on chromatin.

## 6. Proteolytic or Non-Proteolytic Functions of the Proteasome?

One of the other major controversies in this field has been the role that proteolytic *versus* non-proteolytic functions of the proteasome play in the regulation of transcription. This controversy has been fueled in part by debate over whether 26S proteasomes are the transcriptionally-relevant species, or whether distinct 19S subassemblies—lacking proteolytic function—are at work on chromatin [24,51], and by differences in how transcriptional processes respond to inhibition of 19S *versus* 20S proteasome function (e.g., [52,53,54,55]). We have discussed this controversy, and its possible causes, previously [1,55,56,57]. Rather than recount these issues, it is perhaps more instructive to make some generalizations that will assist the reader in later discussions of proteasome function at chromatin.

First, there is compelling evidence that functions of the 19S complex—notably its ATP-dependent chaperone actions—can act independently of proteolysis to control steps in transcription. At the same time, there is equally compelling evidence that 20S proteolytic functions are required for certain events on chromatin, indicating that across the spectrum of transcriptional activities, the full suite of proteasome functions is likely to be involved. Second, even if 19S ATPases act non-proteolytically in this setting, there is no conceptual need to evoke the presence of a distinct 19S subassembly for this to occur. ATP-dependent protein remodeling by the 19S ATPases occurs on the exposed surface of the 19S ATPase ring [58], and 26S proteasomes can separate protein complexes without destroying them [59], meaning that 26S proteasomes likely have all of the requisite chaperone functions of their 19S components. Finally, there are likely to be cases where the distinction between 19S *versus* 20S activities is semantic, and reflects the importance of short *versus* long-term regulation, rather than relevance of a specific function in driving a transcriptional process forward. Theoretically, any ubiquitylated protein delivered to the proteasome will be unfolded—and inactivated—by 19S ATPases ahead of permanent destruction by 20S proteases. In this way, 19S proteins can be seen as mediating the primary point of biological regulation. Whether a 19S or 20S functions scores as more important, therefore, could simply reflect whether the substrate can be refolded and reused, or whether it is shuttled to the 20S for destruction and hence sustained regulation. In the following sections of this review, we will describe known or inferred functions of the proteasome in chromatin-dependent processes, noting whether 19S or 26S functions are implicated but with the assumptions that 26S proteasomes are the relevant species and that distinctions between proteolytic *versus* non-proteolytic activities are not always clear cut or necessarily meaningful.

## 7. Functions of the Proteasome in mRNA-Type Transcription

**Figure 3:**
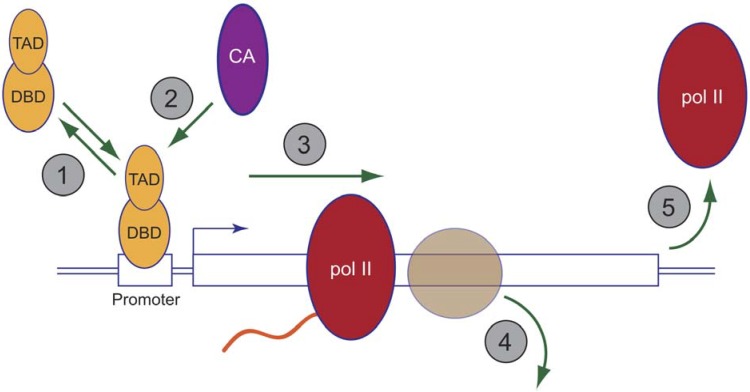
Functions of the proteasome in pol II-mediated transcription. The cartoon shows an idealized mRNA-type gene, with steps shown to be influenced by proteasome function numbered in grey circles 1–5. 1: Controlling the dynamics of activator binding; 2: Controlling co-activator recruitment; 3: Promoting transcriptional elongation; 4: Histone eviction ahead of traveling pol II; 5: Transcription termination.

Transcription is a complex process that involves the action of hundreds of proteins which must function together, and in a highly coordinated way, to facilitate production (in the case of pol II) of a functional messenger RNA. At a basic level, the events of transcription can be broken down into three crude steps—initiation, elongation, and termination—each of which is governed by select groups of ancillary proteins that associate with pol II and usher it through the temporally distinct steps in RNA synthesis. Proteasomes have been implicated in all three phases of transcription (Figure 3).

Traditionally, much of the regulation of gene expression has been thought to occur at the level of transcription initiation. Although this view may be an oversimplification [60], it is clear that transcription initiation is a highly regulated process, and that proteasomes act at several key points to control where and how genes are turned on. The aforementioned enrichment of degradative ubiquitylation (and 20S β1 proteasome subunit binding) immediately upstream of the transcription start site of active genes in mammalian cells [27] points to a general role for proteasomal proteolysis in controlling events at the 5' ends of genes (including initiation), and more focused studies have indicated that several key factors are targeted by proteasomes to control initiation. One key point of control occurs at the level of activators, DNA-binding proteins that sit in 5' gene regulatory regions and act to recruit proteins that control the initiation step.

Interestingly, activators have been reported to be controlled by the proteasome at both the proteolytic and non-proteolytic levels [1], and controversy still lingers regarding which functions are at work, even for the same activator [54]. In the early 1990s, Johnston and colleagues identified mutations in 19S base subunits Rpt4 and Rpt6 as allele-specific suppressors of a near complete deletion of the Gal4 activation domain [18,61,62,63], and published a series of papers culminating with the model that 19S base ATPases act non-proteolytically to strip Gal4 from chromatin, a process that is selectively blocked by activator mono-ubiquitylation [64]. In contrast, a fairly strong case can be made that many activators, including Gal4 [55,65], require *in situ* proteasomal degradation to be active [1], and we have proposed that this requirement for proteolysis reflects the need for cycling of activators on and off target gene chromatin. Although resolving these competing models is not possible at the moment, it is interesting to note that most observations point to a role for Ub and proteasomes in controlling the dynamic association of activators with their cognate DNA elements. This is likely to be an evolutionarily conserved phenomenon, as proteasome function controls the rapid exchange of glucocorticoid receptors on their promoter DNAs in mammalian cells [66], presumably providing a mechanism to fine-tune transcriptional output. Exactly how this occurs will require further experimentation.

In order to function, activators require an additional set of proteins—co-activators—that do not generally signal gene-specific regulation but rather are recruited by activators to facilitate gene induction. One of the clearest examples of how proteasomes control co-activators centers on SAGA, a multi-functional co-activator complex that has multiple enzymatic activities, including histone acetylation [67]. Workman and colleagues found that purified 19S proteasomes use the energy of ATP hydrolysis to alter SAGA, promoting its interaction with DNA-bound activators such as Gal4 [68]. Consistent with this action, mutations in 19S components reduce recruitment of SAGA to target genes *in vivo*, demonstrating that 19S plays a direct role in modulating co-activator recruitment as part of the activation process. What is compelling about this study is that the actions of 19S proteasomes on SAGA were fully recapitulated *in vitro* with highly-purified components, meaning that the actions of 19S on SAGA can unambiguously be defined as non-proteolytic. The most likely scenario is that 19S base proteins function as ATP-dependent chaperones or translocases, selectively remodeling one or more subunits of SAGA to increase its affinity for activator proteins (see Section 8 for a description of one documented function of 19S on SAGA subunit composition). The stringent demonstration that 19S proteins act non-proteolytically in this capacity provides compelling support for the notion that multiple functions of the proteasome are at work in transcription. A vital outstanding question, however, is whether this action of 19S on SAGA can be exerted by intact 26S proteasomes, or whether some kind of transient separation of 19S and 20S complexes is needed for these non-proteolytic activities. Indeed, the defined biochemical nature of the systems used in this study would seem a perfect venue to resolve the question of whether, as we speculate [56], 26S proteasomes are capable of acting as transcription complex chaperones, without the need to dissociate into distinct subassemblies.

After initiation, pol II must elongate across the template to synthesize a full-length mRNA. Post-initiation steps such as these are difficult to study in situations where proteasomes are also involved in initiation, but there is compelling data to believe that both elongation and termination are under the control of proteasome-dependent processes. Indeed, in our mapping of proteasome subunit distribution on active genes [21] we found that the greatest density of proteasome subunits in the *GAL10* and *PMA1* genes in yeast is within the transcribed portion of the gene, implying a role for the proteasome in steps downstream of initiation. If such functions do exist, they are likely to be non-essential or non-proteolytic, because comprehensive proteasome inhibition does not universally impede transcription, even of genes with fairly high proteasome density [69]. Further supporting non-proteolytic actions of the proteasome in elongation, mutations in 19S ATPases induce transcriptional defects *in vivo* and in crude *in vitro* systems [70]. Reed and colleagues developed this concept further by showing that the 19S ATPases are recruited to the PAF transcriptional elongation complex via an adapter protein called Cks1 [71], and that contact between the 19S subunit Rpt1 and PAF is required for efficient transcriptional elongation. Coupled with their findings that mutations in 19S genes impair the ability of cells to evict nucleosomes ahead of traversing pol II [46], Reed and co-workers conclude that a primary point of action of 19S proteasome components in elongation is the ATP-dependent, chaperone-like, removal of histones from template DNA, allowing pol II an unimpeded path for gene transcription. Rigorous biochemical support for such a “histone chaperone” function of the proteasome is yet to come, however, and the one study that directly addressed this issue concluded that 19S and 26S proteasomes do not posses inherent ATP-dependent chromatin remodeling activity [72]. It is certainly possible that some essential component of the chromatin remodeling process is missing from *in vitro* systems (e.g., a ubiquitylated substrate), but discrepancies such as these also serve as a useful reminder of the difficulty in assigning specific functions of the proteasome in transcription, given its ubiquitous role in controlling the expression of thousands of proteins in the cell.

Finally, to ensure the integrity of transcripts, and to prevent interference with transcription of neighboring genes, pol II must terminate transcription at a fairly specific point or set of points at the 3' end of the gene. The interaction of 19S proteins with the PAF complex could certainly influence transcriptional termination, as PAF has multiple actions in transcription, including 3'-end formation at polyadenylated and non-polyadenylated transcripts [73,74]. Although this issue has not been looked at exhaustively, one study has found that inhibiting the proteolytic activity of the proteasome causes pol II to “read through” normal sites of transcriptional termination at specially-engineered reporter genes [42]. This finding reveals that something needs to be destroyed by the proteasome in order for pol II to terminate properly. But what? It is highly unlikely that pol II itself is simply destroyed by the proteasome at the end of transcription. Rather, it is more reasonable to speculate that some ancillary protein that is present in complex with pol II is the target for degradation, perhaps one that acts to prevent ectopic termination of transcription within the body of the transcription unit.

Although this brief discussion focused on transcription by pol II, it should be stressed that components associate robustly with genes transcribed by RNA polymerases I [75] and III [21], and that at least in the case of RNA polymerase I, pre-ribosomal RNA synthesis is blocked by chemical inhibition of 20S protease activity [75]. It appears as though all three nuclear RNA polymerases have evolved to recruit proteasomes for one or more tasks important to their functions.

## 8. The Proteasome and Chromatin Modifiers

We have already discussed the proposed role of 19S ATPases acting to evict nucleosomes ahead of pol II, but given the widespread association of proteasomes with chromatin it is likely that other actions of the proteasome on histones or their modifiers exist. There is little published evidence that proteasomes destroy chromatin-bound histones, except during spermatogenesis (above), but there are examples of non-proteolytic actions of proteasomes on histone modifiers. We found, for example, that mutations in 19S ATPases lead to a loss of methylation of histone H3 at lysines 4 (H3K4) and 79 (H3K79) [22], modifications that occur on transcriptionally-active chromatin but paradoxically are required for subtelomeric gene silencing, most probably because these marks are repelling components of the Sir silencing complex, keeping them localized to telomeres. Accordingly, mutations in Rpt4 and Rpt6 result in a loss of telomeric gene silencing [22], although we do not know whether this is solely due to a loss in H3K4/K79 methylation, or whether the recently discovered binding of proteasomes to telomeres [21] reflects an active role for proteasomes in repressing transcription at telomeric DNA.

As discussed above, 19S proteasomes are capable of somehow remodeling SAGA to promote efficient interaction with DNA-bound activators [68]. Precisely how this occurs is unknown, but in following up these studies the Lee group showed one way in which 19S proteins remodel SAGA [76]. SAGA is a multisubunit complex that can be divided into four distinct subassemblies, including a dUb module that most prominently removes mono-Ub from histone H2B [67]. Lee and co-workers showed that association between 19S is mediated by an interaction between the 19S base ATPase Rpt2 and the SAGA component Sgf73, and that this association leads to the ATP-dependent ejection of critical parts of the dUb module from SAGA. Importantly, the ejected dUb module retains its enzymatic activity, suggesting that 19S-mediated remodeling of SAGA frees the dUb component for some specific function, perhaps at a time or stage in transcription where the rest of the SAGA complex is required elsewhere. Consistent with this notion, mutations in Rpt2 alter distribution of the dUb module on active chromatin, relative to the remainder of the SAGA complex. Moreover, these mutations also block the established function of Sgf73 in promoting association of active genes with nuclear pore complexes and facilitating mRNA export [77]. These results provide compelling evidence that non-proteolytic functions of 19S proteasomes act to remodel SAGA to drive the timing of distinct steps in gene expression.

Another striking link between the proteasome and chromatin modifiers was discovered by the Conaway laboratory, who reported that the Uch37 deubiquitylating enzyme (dUb) that is part of the 19S regulatory particle and clips Ub chains from proteins before they enter the proteasome [78]—is a bonafide member of the Ino80 chromatin remodeling complex [79]. The function of Ino80 is to catalyze ATP-dependent sliding of nucleosomes, permitting access of DNA to proteins involved in events such as transcription [80] and DNA repair [81]. Intriguingly, within the context of Ino80, the dUb function of Uch37 is profoundly inhibited, but its activity can be transiently activated by interaction of Ino80 with proteasomes. The presence of a Uch37 in both the 19S proteasome and a chromatin remodeling complex provides a frank demonstration of connectivity between the transcription and Ub-proteasome systems, and raises an intriguing possibility: If the dUb function of Ino80 is required for chromatin modification, it is likely that it would only be active in the presence of chromatin-bound proteasomes. This is an interesting concept, as it implies that one function of proteasome recruitment to chromatin may be to regulate the activity of complexes such as Ino80. Moreover, this activity appears to require simple physical association between the complexes (mediated via Rpn13; [79]), meaning that its significance would be completely overlooked by standard genetic and chemical approaches to interrogate proteasome function by blocking its proteolytic or ATP-dependent functions. It may very well be, therefore, that there is an entire suite of proteasome functions in transcription and other chromatin-based events that we have yet to uncover.

## 9. Cryptic Transcription

Textbook views of transcription typically paint a picture of a precise molecular process that is highly-specific in terms of which segments of DNA are transcribed and when. Recent studies of the transcriptome, however, have revealed that transcription is a highly pervasive process, and that much of the genome is abuzz with transcription, a large percentage of which produces RNAs that are rapidly destroyed [82]. The process of cryptic transcription fits into this category. As its name implies, cryptic transcription occurs when RNA polymerase synthesizes an RNA from a non-genic region of the genome, and happens, for example, when pol II transcribes in the “opposite” direction from an authentic promoter element, or when nucleosome-depleted regions of the genome expose non-authentic promoters that are capable of recruiting the transcription machinery [83]. Little is known of how the proteasome influences this process, but it is interesting to note that mutations in proteasome subunits have been uncovered in genetic screens for factors that repress cryptic transcription [84]. Moreover, in mouse embryonic stem cells, genetic or chemical inhibition of proteasome function leads to an increase in cryptic transcription from select loci [35]. In this study, the authors showed that both 19S and 20S proteasome subunits are recruited to sites of cryptic transcription, implying that the function of the proteasome in this context is to destroy either RNA polymerase or pre-initiation complex members that drive transcription at these ectopic sites. The notion of proteasomes promoting transcriptome integrity by destroying inappropriate transcription complexes is intuitively aligned with how proteasomes feature in other biological processes, such as protein quality control, and clearly warrants further investigation to determine its scope and underlying mechanisms.

## 10. Conclusions and Perspectives

All available evidence points to an extensive role for proteasomes in the control of nuclear events, particularly those that occur in the context of chromatin. It is also clear that proteasomes can act on chromatin as canonical proteases and non-canonically as protein chaperones. What is generally missing in this field, however, are precise molecular details regarding the substrates that are involved, whether they are destroyed or non-proteolytically remodeled, and the characterization of the impact of proteasome action on specific stages in chromatin expression or refurbishment. As mentioned, the general involvement of proteasomes in protein homeostasis can make it very difficult to draw robust molecular conclusions from results of genetic or chemical inhibition of proteasome function. Fortunately, the expanding number of chromatin-centric events in which the proteasome is implicated, increases the likelihood that more substrates and processes will be pinpointed in the near future, facilitating the development of biochemical reconstitution experiments that will be necessary to delineate underlying mechanisms. This process will be greatly facilitated by the maturation of the Ub-proteasome field itself, and increasing knowledge of its mechanisms, generalities, and nuances.
